# Water-Spray-Cooled Quasi-Isothermal Compression Method: Water-Spray Flow Improvement

**DOI:** 10.3390/e23060724

**Published:** 2021-06-06

**Authors:** Guanwei Jia, Xuanwei Nian, Weiqing Xu, Yan Shi, Maolin Cai

**Affiliations:** 1 School of Physics and Electronics, Henan University, Kaifeng 475004, China; jiaguanwei@buaa.edu.cn; 2 School of Automation Science and Electrical Engineering, Beihang University, Beijing 100191, China; nianxuanwei218@buaa.edu.cn (X.N.); shiyan@buaa.edu.cn (Y.S.); caimaolin@buaa.edu.cn (M.C.); 3 Pneumatic and Thermodynamic Energy Storage and Supply Beijing Key Laboratory, Beijing 100191, China

**Keywords:** water-spray cooling, flow improvement, quasi-isothermal compression, heat transfer, compressed-air energy storage

## Abstract

Water-spray-cooled quasi-isothermal compressed air energy storage aims to avoid heat energy losses from advanced adiabatic compressed-air energy storage (AA-CAES). The compression efficiency increases with injection water spray. However, the energy-generated water spray cannot be ignored. As the air pressure increases, the work done by the piston and the work converted into heat rise gradually in the compression process. Accordingly, the flow rate of the water needed for heat transfer is not a constant with respect to time. To match the rising compression heat, a time sequence of water-spray flow rate is constructed, and the algorithm is designed. Real-time water-spray flow rate is calculated according to the difference between the compression power and heat-transfer power. Compared with the uniform flow rate of water spray, energy consumption from the improved flow rate is reduced.

## 1. Introduction

The greatest demand of global electrical energy (>70%) is met by burning traditional fossil resources. The results have led to huge carbon dioxide (CO_2_) emissions and global warming, threatening human survival [1]. The development of renewable energies (such as wind, solar, etc.) is often advocated as an effective way to meet the demand for electrical energy and reduce CO_2_ emissions [2]. For this reason, renewable energies have been rapidly developed at a global scale [3]. Nonetheless, the intermittency and fluctuation of renewable resources create obstacles and challenges to accessing to the electrical grid [4]. Large-scale energy storage for stability and security of the energy supply is recognized as an efficient resolution to the obstacles [5]. The compressed-air energy-storage (CAES) system can provide a larger energy-storage scale (100 MWh, battery <10 MWh), higher environmental friendliness (no heavy metal pollution), and longer service life (20–60 years; battery <20 years) [6]. However, the round-trip efficiency (RTE) of traditional CAES is lower (<50% [7]) which makes large-scale applications more difficult [8,9]. A water-spray-cooled quasi-isothermal CAES system can provide a higher efficiency without CO_2_ emissions [10].

Water-spray-cooled isothermal compression technology employs a water pump to generate high-pressure water, which is atomized by the nozzle to form a water spray. Then the water spray is injected into the compression chamber to increase the heat-transfer area so that the water spray can be fully mixed with the air and absorb the compression heat [11]. Currently, significant attention has been paid by researchers to methods of enhancing the heat transfer between the water spray and air for reduction of the total compression work. Coney [12] injected water sprays into the compression chamber to cool the compressed air. The experimental results demonstrated that the temperature of the compressed air was less than 100 °C and the compression work was reduced by 28% at a compression ratio of 25 and a speed of 380 rpm. Patil et al. [13] achieved near-isothermal compression using water-spray injection with a compression ratio of 2.5, an injection pressure of 483 kPa, and a spray-nozzle angle of 60°. The experimental isothermal efficiency was increased by 4‰. The SustainX Company [14] upgraded the nozzles and the spray generation systems for obtaining a water spray diameter of 100–900 μm. Their experimental results showed that the compression efficiency was more than 95%, and the generated water-spray work was less than 2% of total system work. The LightSail Company [15] injected a water-spray into the compression chamber to absorb and store the compression heat. The temperature rise of the compressed air was less than 10 °C, the compression efficiency reached 90%, and the compression/expansion cycle efficiency reached 70%. Jia et al. [16] experimentally obtained a water-spray-cooled isothermal compression efficiency of 92% using a water-spray diameter of 10–100 μm, a flow rate of 0.416g/cycle, and a compression volume ratio of 2.5. Odukomaiya et al. [17] employed a water-spray diameter of 50 μm to boost the efficiency to 96% and achieve a roundtrip efficiency of 70% at a water-flow rate of 12 L/min. Chen et al. [18] applied a water-spray diameter of 50 μm to achieve a temperature rise as low as 5 °C and an efficiency as high as 98% at a water-flow rate of 10 L/min using open isothermal CAES. Qin et al. [19] numerically studied a water-spray diameter of 30.6 μm in the first-stage compression chamber and found that the compression efficiency was improved to as high as 92% at a mass loading of 1.6. Heidar et al. [20] employed a water-spray diameter of 30 μm to inject the compressor at a flow rate of 4 kg/s based the Huntorf plant’s operating and technical information. The simulation results showed that the roundtrip efficiency was up to 42.64%, and improved 2.5% (see [10]). Qin [21] et al. investigated a water-spray diameter of 20 μm to inject the compressor with a mass loading of 5 and a compression ratio of 10. The simulation results presented a near isothermal compression efficiency of up to 92%. Dib et al. [22] established mathematical models of three air compressor/expander stages with a water-spray diameter of 20 μm to achieve quasi-compression/expansion. The results showed that the air temperature gradient was nearly constant (1 °C) from the outlets of three stages, and quasi-compression efficiency reached 64% at a mass loading of 5.

Given the thorough literature review, a micron-level water spray is necessary for achieving quasi-isothermal compression and expansion. Water must be forced by high pressure through a small nozzle with high speed. The friction between the water and the air disrupts the water steam into water sprays. The energy generated by the pressure difference that the water pump consumes depends on the product of the flow rate and the pressure difference. Table 1 shows the ratios of pump energy consumption to compressor energy consumption used in previous studies. Thus, the energy consumption by the pump is required. The flow rate of the water spray especially affects the water pump.

As the air pressure increases in the compression process, the work done by the piston and the heat generated from air compression rise gradually. A constant flow rate applied in the studies does not equal the rising heat-transfer requirement. In this paper, we propose a method to improve the flow rate of the water spray by matching it to the heat from compression.

## 2. Mathematical Model

To simplify the calculation, the assumptions are as follows:

(1) The water-spray is assumed to be evenly distributed in the compression chamber;
(2) Heat transfer between the water on the surface of the piston and the air is ignored;
(3) The external forces on the motion of the water spray have been not taken into account;
(4) The air is treated as an ideal gas.

### 2.1. Air Temperature and Air Pressure

**Figure 1:**
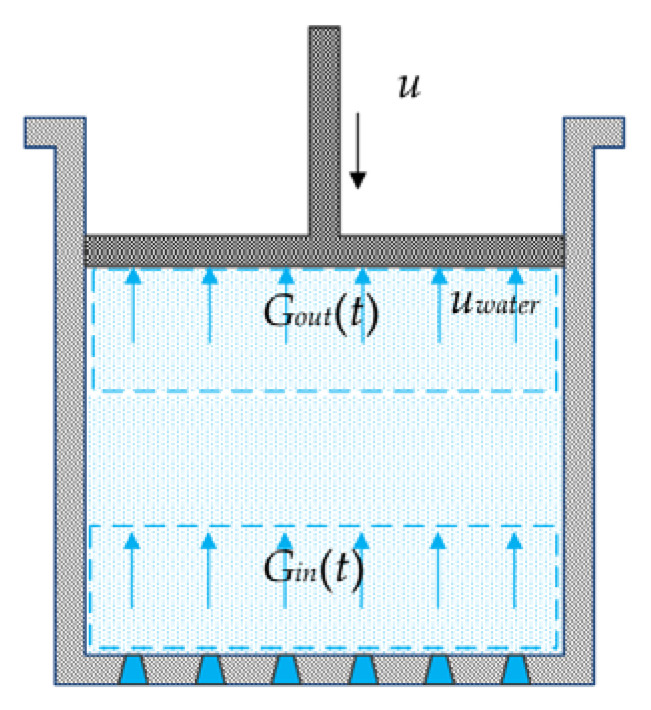
Movement of the water spray in the compression chamber.

In the gas-compression process, the movement of the water spray and its distribution are complicated. To simplify the calculation, the water spray is assumed to be evenly distributed in the compression chamber, and the influences of the uneven distribution of water spray on the temperature and the pressure are ignored. A multinozzle configuration was needed in this study, as shown in Figure 1. If the size of the nozzle approaches zero and number of the nozzles approaches infinity, the even distribution approximation can be achieved. The average temperature was investigated in this study.

According to the law of conservation of energy, the heat transferred to the compressed air (*δQ*) and the work the piston does on the air (−*dW*) lead to an increase in the internal energy (*dU*):

(1) dU=δQ−dW

According to the definition of specific heat (*c*), the relationship between internal energy and temperature (*dT*) can be established as:

(2) dU=m⋅c⋅dT

According to the ideal gas state equation, the gas pressure can be expressed as a function of temperature and volume, which is obtained by differentiating the ideal gas state equation. The reason for using the ideal gas is that under various conditions of temperature and pressure, many real gases behave qualitatively like an ideal gas, where the gas molecules (or atoms for a monatomic gas) play the role of the ideal particles. Air can be treated as ideal gases within reasonable tolerances [23]:

(3) Vdp+pdV=mRdT

### 2.2. Heat Transfer

According to Newton’s law of cooling, the heat transfer between object 1 and object 2 is proportional to the heat transfer area S and the temperature difference between the two objects:

(4) $$\delta Q=hS\left(T_{1}-T_{2}\right)dt$$

where *h* is the heat transfer rate.

### 2.3. Flow Mass and Heat-Transfer Area of the Water Spray in the Compression Chamber

The movement of the water spray in the compressor chamber is shown in Figure 1. The water spray was spread from the nozzles at the bottom of the compression chamber. The water-spray flow rate at the nozzles was defined as *G_in_*(*t*). The water spray flowed through the compression chamber until it was collected on the lower surface of the piston. The heat-transfer area of the air was not improved by the water on the surface of the piston, as its area could not be greater than that of the piston. For example, with a water-spray diameter of 19.6 μm and 38.5 μm, the lower surface of the piston only accounted for 3.71% and 7.29%, respectively, of the total surface of the water spray. Therefore, the heat transfer between the water on the surface of the piston and the air was ignored in the following discussion. The water-spray flow rate collected on the piston was defined as *G_out_*(*t*). The net flow rate of the water spray in the compression chamber was defined as *G*(*t*):

(5) $$G(t)=G_{in}\left(t\right)-G_{out}\left(t\right)$$

The motion of water spray in the compression chamber was affected by gravity and air resistance. To simplify the calculation, the influence of external forces on the motion of the water spray was ignored in this study. The compressor and simulation parameters used in the calculation are shown in Table 1, Table 2 and Table 3 for a compression chamber length of 0.12 m, an average diameter of the water spray of 19.6–38.5 μm, and a water-spray speed of 0.4 m/s. The external forces on the motion of the water spray mainly included gravity and air resistance. The motion of the water spray would present a challenge when combining the external forces with the length of the compression chamber because the motion of the water spray was influenced by the parameters of initial velocity, acceleration, distance, and air-resistance coefficient. Therefore, the motion of the water spray was considered as an average velocity to simplify the calculation, such as 0.4 m/s. Then, the relationship between *G_in_*(*t*) and *G_out_*(*t*) was obtained:

(6) $$G_{out}(t)=\alpha G_{in}\left(t-\tau \right)$$

where *τ* is denoted as the time required for the water spray to flow between the nozzles and the surface of the piston. *L*_0_ denotes the piston stroke.

(7) $$\tau =\frac{L_{0}}{u_{water}+u}$$

where *α* represents the influence of the piston velocity (*u*) on the amplitude of *G_out_*(*t*). Increasing the speed of the piston increases the flow to the lower surface of the piston.

(8) $$\alpha =\frac{u_{water}+u}{u_{water}}$$

**Figure 2:**
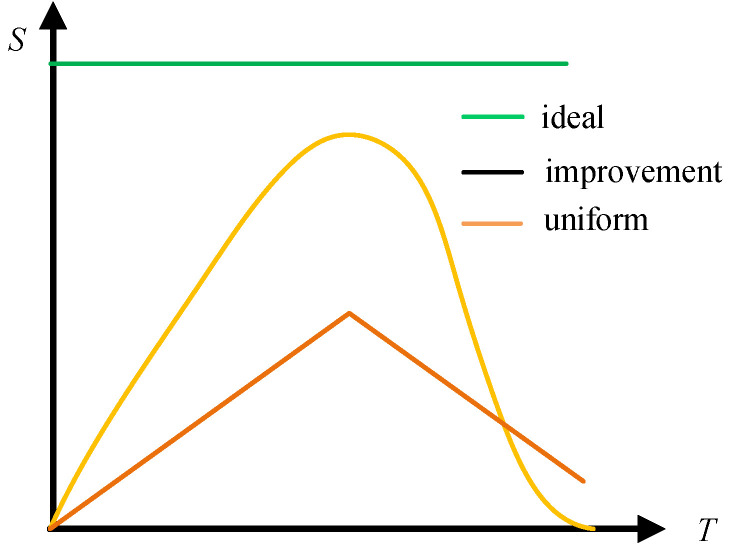
Change of heat-transfer area during compression.

The cumulative mass of water spray in the compression chamber can be solved according to the integral of Equation (5), and the heat transfer area is proportional to the cumulative mass of the water spray. As shown in Figure 2, the “uniform” curve indicates that the water-spray heat-transfer area in the compression chamber changes linearly with time when the water spray flow is constant. The initial stage of compression rises linearly, and the later stage of compression declines linearly. The “improvement” curve indicates that the heat-transfer area changes nonlinearly with time after the water-spray flow is improved, and the following equation can be obtained, where *S*(*t*) is the heat-transfer area between the water spray with the compressed air in compression chamber, m^2^:

(9) $$S\left(t\right)=\frac{\int_{0}^{t}\left[G_{in}\left(t\right)-G_{out}\left(t\right)\right]dt}{\rho_{water}}\cdot \frac{6}{d}$$

To indicate the limit of the heat-transfer area that can be achieved by consuming a given mass of water spray, an ideal condition (“ideal” curve) is defined, which means that the heat-transfer area is fully utilized in the compression process, and the heat-transfer area is always equal to the total surface area of the water spray consumed. *S_ideal_*(*t*) was defined as the consuming of a given mass of water spray with full utilization, m^2^:

(10) $$S_{ideal}\equiv \frac{m_{in}}{\rho_{water}}\cdot \frac{6}{d}$$

where *d* is the diameter of water spray, *m*.

### 2.4. Power Consumption of the System

The system power consumption (*W_+water_*) discussed in this paper was composed of two parts: the power consumption of the piston working on air (the power consumption of compression) and the power consumption of the water pump when the water-spray is generated (the power consumption of atomization).

(11) $$W_{+water}=W+W_{water}$$

#### 2.4.1. Power Consumption of Compression

During the compression process, the piston maintained a constant speed, and the external driving force of the piston was in equilibrium with the pressure of the air in the compression chamber. Then, the compression work (*W*) is:

(12) $$W=-\int_{V_{0}}^{V}pdV$$

#### 2.4.2. Power Consumption of Atomization

The condition under which the water spray was generated is the high-speed water flow; that is, the generation of the water spray requires the kinetic energy of the water flow, which is defined as the power consumption of the atomization. In the water-spray system, the work of the high-pressure water pump was converted into kinetic energy of the water flow through the nozzle to increase the speed of the water flow. Therefore, the atomization power was derived from the work done by the pump, which can be expressed as the product of the pump pressure and the flow volume:

(13) $$W_{water}=p_{water}\cdot V_{water}$$

#### 2.4.3. Ideal Total Power Consumption

The ideal conditions for this study included the heat-transfer area defined in Section 2.2, and the water spray and air having sufficient heat transfer and reaching thermal equilibrium at equal temperatures. Under ideal conditions, the heat-absorption capacity of the water spray is fully utilized, and the heat-transfer coefficient is large enough. The energy equation can be obtained by applying Equation (1):

(14) $$\left(C_{v}m+C_{water}m_{water}\right)dT=-pdV$$

In conjunction with the ideal gas state Equation (3), the pressure can be obtained by integrating the volume, and the total system power consumption can be obtained by substituting Equation (11) and Equation (12):

(15) $$W_{+water}=p_{0}V_{0}\cdot \frac{{\left(V_{0}/V\right)}^{n-1}-1}{n-1}+W_{water}$$

where $n=\frac{C_{p}m+C_{water}m_{water}}{C_{v}m+C_{water}m_{water}}$.

## 3. Methods

### 3.1. Objective Function

The goal of improvement was to minimize *W_+water_*, the sum of compression work and atomized water-spray work. The factors that affected the total power consumption were as follows:

The water-spray flow affected the water mass and heat-transfer area in the compression chamber. Increasing the water-spray flow increased the heat-transfer area and reduced the compression power. However, the power consumption required to atomize the water spray increased.

Since the pressure in the compression chamber and the heat generated by the compression vary with time, the flow of water spray required for cooling should also change with time.

According to the analysis in Section 2.3, the movement of the piston reduced the water spray heat transfer time. The water sprayed into the compression chamber in the early stage had a longer heat-transfer time than the water sprayed later.

Therefore, the amplitude and time-domain characteristics of the water-spray flow affected the total power consumption. The improvement discussed in this paper refers to constructing the water-spray flow function *G*(*t*) to reduce the total energy consumption.

### 3.2. Improvement Principle

The piston work is converted into internal energy of the air and heat absorbed by the water spray. Based on the difference indicating the degree of deviation of the compression process from isothermal compression between the compression work and the heat absorbed by the water spray, the increment of the water spray flow at each moment can be constructed as shown in Equation (16). The greater the difference is, the greater the demand for water-spray flow. This leads to an increase in the flow of water spray. When the difference value approaches zero, the compression process approaches isothermal compression. Then it is not necessary to increase the water-spray flow.

(16) $$\Delta G_{in}\left(t\right)\propto dW\left(t\right)-\delta Q\left(t\right)$$

Energy conservation Equation (1) is applied to obtain:

(17) $$\Delta G_{in}\left(t\right)=\frac{puA-\left[C_{water}\cdot G_{in}\cdot \left(T_{water}-T_{water0}\right)+C_{water}\cdot m_{in}\cdot {\dot{T}}_{water}\right]}{C_{water}\cdot \left(T_{water}-T_{water0}\right)}$$

### 3.3. Algorithm

**Figure 3:**
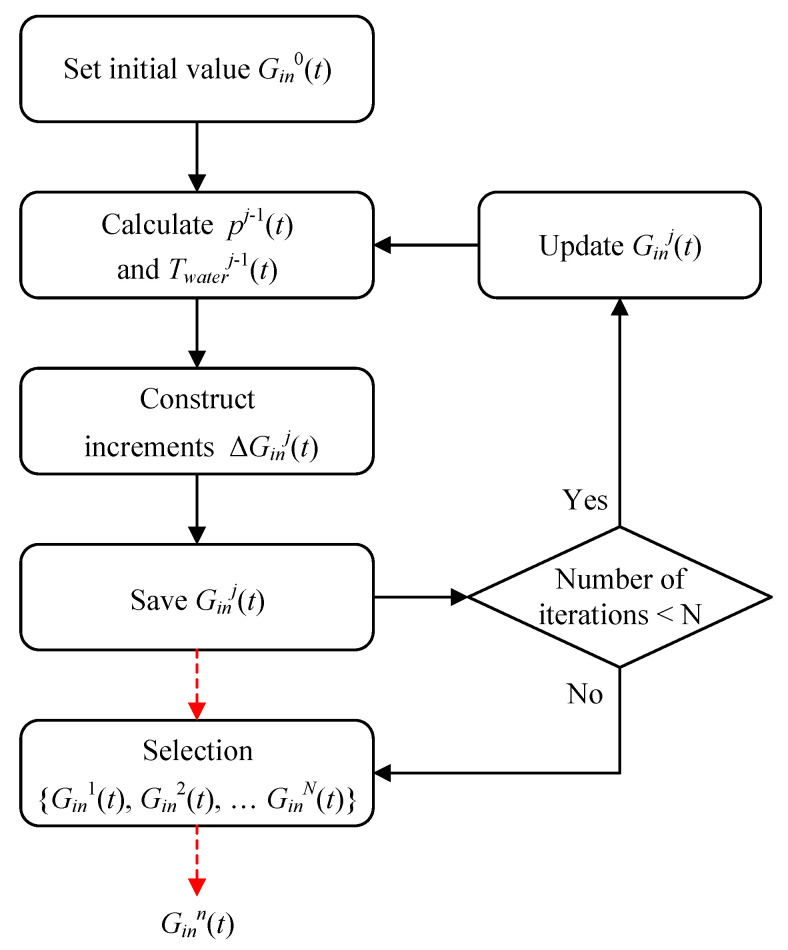
Architecture of the algorithm.

The algorithm was divided into two parts: construction and selection. The first was used to construct multiple sets of water-spray flow functions so that the compression process approached isothermal compression; the second was used to select a set of water-spray flow functions to minimize the total energy consumption. The specific steps are shown in Figure 3.

The initial estimation of water spray flow was set as: $G_{in}{}^{0}\left(t\right)=0$.

The state of air and water spray were solved based on the compressor model. The estimated value of the flow $G_{in}{}^{0}\left(t\right)$ was substituted into the compressor model to calculate the air pressure $p^{j-1}\left(t\right)$ and the water-spray temperature $T_{water}{}^{j-1}\left(t\right)$.

The increment of the flow rate $\Delta G_{in}{}^{j}\left(t\right)$ was calculated according to Equation (17). The compression cycle was divided into multiple intervals with a time length of ∆t. Within the *j*-th iteration and the *i*-th interval, the flow was represented by $G_{in\;i}{}^{j}$. According to Equation (17), the parameter relation between the two iterations could then be obtained:

(18) $$\Delta G_{in\;i}{}^{j}=\frac{{\left(puA\right)}_{i}^{j-1}-C_{water}\cdot \left(m_{in\;i-1}{}^{j-1}+\Delta m_{in\;i-1}{}^{j}\right)\cdot {\dot{T}}_{water\;i}{}^{j-1}}{C_{water}\cdot \left(T_{water\;i+1}{}^{j-1}-T_{water0}\right)}-G_{in\;i}{}^{j-1}$$

where $\Delta m_{ini-1}{}^{j}$ denotes the difference in the cumulative mass of water-spray between the two iterations.

The water spray flow was updated and saved as:

$$G_{in}{}^{j}\left(t\right)=G_{in}{}^{j-1}\left(t\right)+\Delta G_{in}{}^{j}\left(t\right)$$

Equation (2) was repeated, and j was used to represent the number of iterations. After *N* iterations, a set of constructors $\left\{G_{in}{}^{1}\left(t\right),G_{in}{}^{2}\left(t\right),\;\ldots \ldots ,G_{in}{}^{N}\left(t\right)\right\}$ was obtained, and when N−>∞, isothermal compression was achieved.

A set of water-spray flow curves $G_{in}{}^{j}\left(t\right)$ were selected from the set $\left\{G_{in}{}^{1}\left(t\right),G_{in}{}^{2}\left(t\right),\;\ldots \ldots ,G_{in}{}^{N}\left(t\right)\right\}$ so that the corresponding total power consumption was the smallest in the set $\left\{W_{+water}{}^{1}\left(t\right),W_{+water}{}^{2}\left(t\right),\;\ldots \ldots ,W_{+water}{}^{N}\left(t\right)\right\}$.

### 3.4. Relaxation Factor K

The amplitude of the flow increment indicated the amplitude of the compression process approaching isothermal compression after one iteration. The larger the amplitude was, the larger the amplitude of change of the result (pressure, temperature, and total power consumption) obtained in one iteration. We defined the relaxation factor K to represent the amplitude of the flow increment:

(19) $$\Delta G_{in\;i}{}^{j}=K\cdot \left[\frac{{\left(puA\right)}_{i}^{j-1}-C_{water}\cdot \left(m_{in\;i-1}{}^{j-1}+\Delta m_{in\;i-1}{}^{j}\right)\cdot {\dot{T}}_{water\;i}{}^{j-1}}{C_{water}\cdot \left(T_{water\;i+1}{}^{j-1}-T_{water0}\right)}-G_{in\;i}{}^{j-1}\right]$$

**Figure 4:**
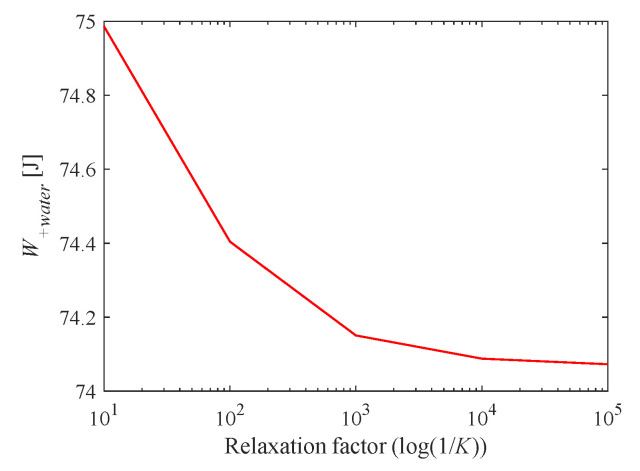
Effect of relaxation factor K on improvement results.

Decreasing the value of *K* could improve the resolution of the constructor and affected the results of this algorithm. Figure 4 shows that the total power consumption ($W_{+water}{}^{n}$) was reduced as K decreased. When *K* was less than 0.001, the total power consumption tended to converge and did not change. Therefore, we choose *K* = 0.001 to be used in subsequent calculations.

### 3.5. Number of Iterations N

**Figure 5:**
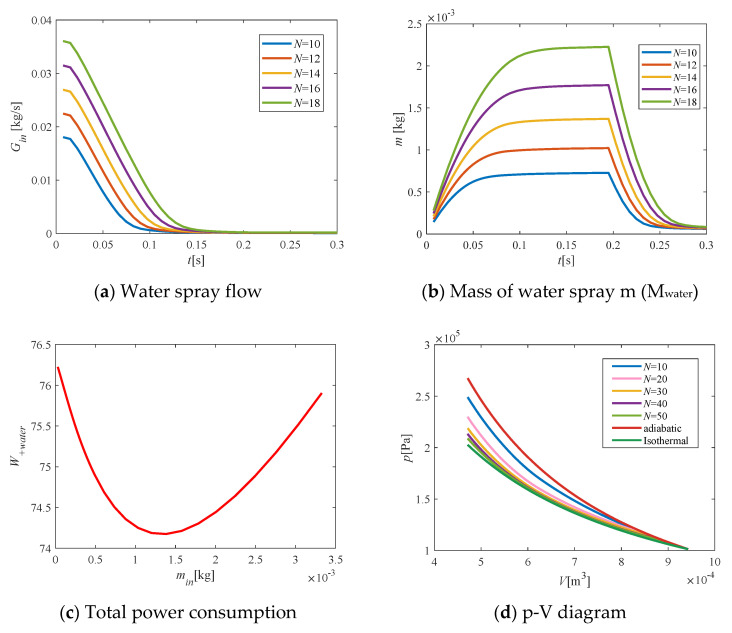
Relationship between the number of iterations N and the flow and total power consumption.

Figure 5a–c shows that as the number of iterations increased, the water-spray flow and mass increased continuously. At each iteration, the increment of the water spray flow was nonuniform in time, and the water-spray flow was large during the initial period and then monotonically decreased. Figure 5d shows that as the cumulative quality of water spray changed, the total power consumption also was nonmonotonic. When the cumulative mass was small, the water spray played a leading role in the energy consumption of compression, and with the increase in the water-spray cumulative mass, the total power decreased. When the mass was large, the increase in water-spray power consumption played a leading role, and with the increase in water-spray cumulative mass, the total power increased.

## 4. Analysis of Results

### 4.1. Total Energy Consumption of the Compression System

The above algorithm was used to obtain the water-spray flow (*G_in_*) and mass (*m_in_*). The difference in total power consumption between the two spray schemes was compared to verify the effectiveness of the algorithm. The compressor parameters used in the calculation are shown in Table 2, the water spray parameters are shown in Table 3, and the other parameters are shown in Table 4.

**Figure 6:**
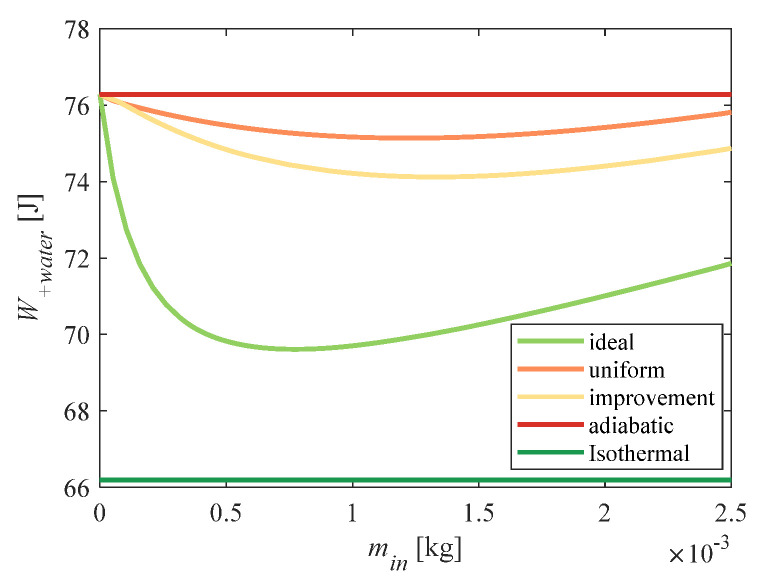
Comparison of total energy consumption.

Figure 6 shows the difference between total power consumption under different water-spray quality conditions. The abscissa indicates the total mass of the water spray consumed. The red curve indicates the relationship between the total power consumption and the mass of the water spray when a uniform flow (constant) water spray was used. The orange curve represents the change in total power consumption when the improved (variable) flow scheme obtained by the above algorithm was used. The blue curve represents the total power consumption under ideal conditions obtained by applying Equation (15). The minimum values of total power consumption corresponding to uniform flow and improved flow were 75.15 J and 74.17 J, respectively, which were 1.7% and 3.2% lower than the total power consumption of adiabatic compression. Therefore, the water-spray flow scheme obtained by the above algorithm was more labor-saving than the uniform scheme. However, there was still a large deviation from the ideal conditions (sufficient heat exchange).

**Figure 7:**
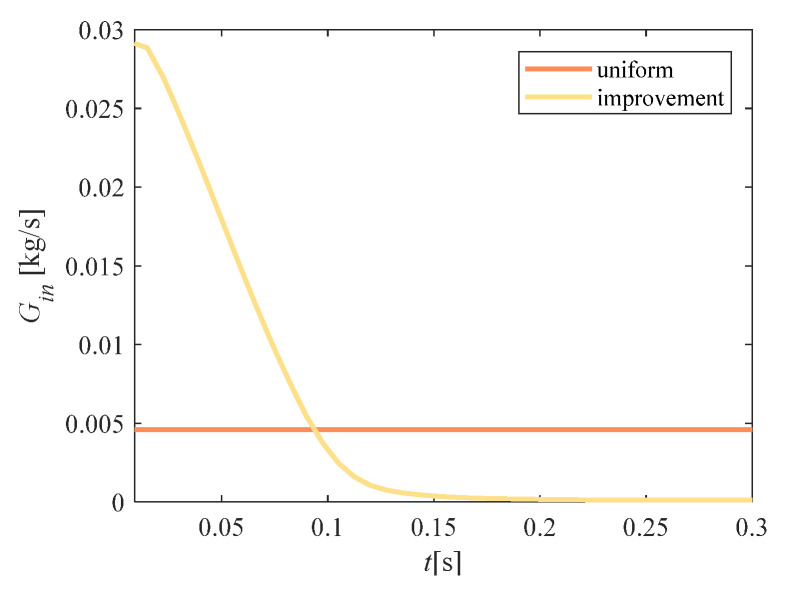
Comparison of flow *G_in_*.

Figure 7 shows the difference between the two schemes. These two curves represent the water-spray flow at the lowest point of total power consumption (points a and b in Figure 6). The flow of the improvement scheme was larger than the uniform flow in the early stage, and tended to be close to 0 in the later stage, while the uniform flow remained constant over time.

**Figure 8:**
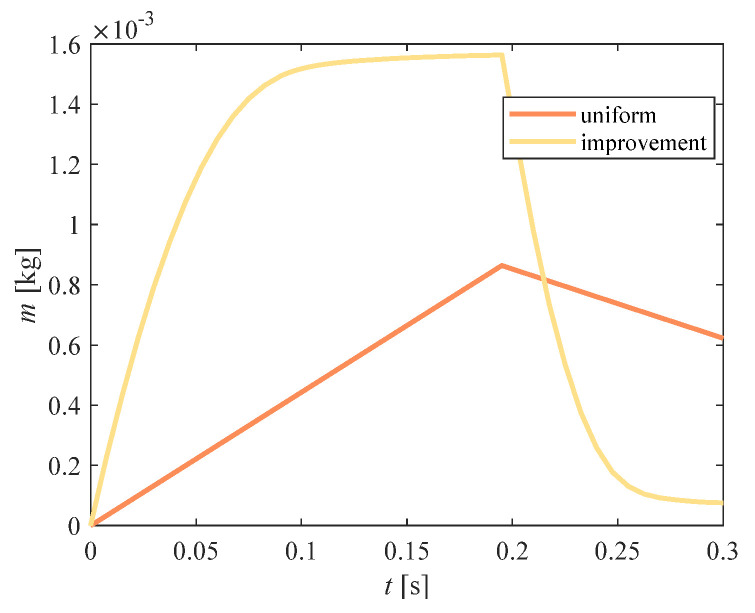
Comparison of cumulative mass in the compression chamber (*M_water_*).

Figure 8 shows the variation in the cumulative water-spray mass in the compression chamber corresponding to the two flow regimes over time. Since the mass was the integral of the flow, the accumulated mass obtained by improvement rose rapidly with a high peak value and approached 0 in the later stage. Under uniform flow conditions, there was still a part of water-spray mass present in the later stage.

**Figure 9:**
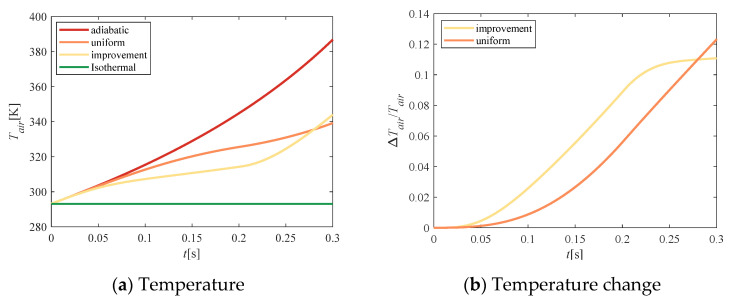
Temperature comparison.

Figure 9 shows the effect of the above cumulative mass on the temperature of the compressed air. As shown in Figure 9a, during the entire compression process, the temperature increment of the adiabatic compression was maximal, and the isothermal compression temperature remained unchanged. After the addition of water spray, the temperature was significantly lower than the adiabatic temperature. The temperature of the improved solution was lower than that of the uniform velocity scheme in the early stage of compression, and was slightly higher in the later stage. In Figure 9b, we compare the difference in temperature between the two methods. The ordinate represents the decline rate of the temperature in the compression process with water spray compared with that of adiabatic compression. The temperature of improved spray water changed quickly, and the amplitude was small, while the temperature of uniform spray water changed slowly and increased continuously.

**Figure 10:**
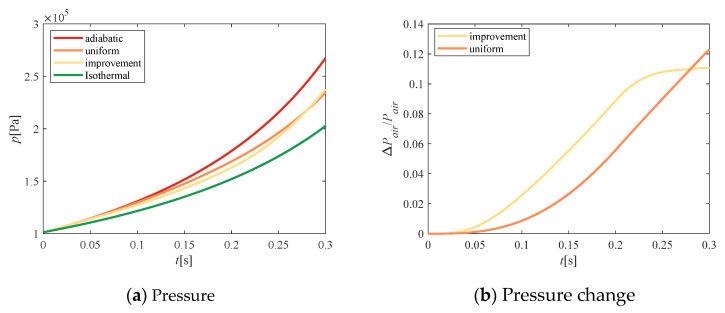
Pressure comparison.

According to the gas state equation, pressure is proportional to temperature, so the pressure change trend shown in Figure 10a was the same as that of temperature. Therefore, the air pressure obtained by the improvement method also starts out smaller than that of the uniform method, and gradually exceeds that of the uniform method over time. Figure 10b shows the response of the pressure change (Δ*p*) caused by water-spray heat transfer. The ordinate indicates the rate of pressure drop compared to that of adiabatic compression. The area enclosed by the curve represents the reduction in compression power consumption (the piston speed was constant). The slope of the response curve of uniform flow increased continuously, presenting a “concave curve”. The slope of the response curve of the improved flow decreased continuously, showing a “convex curve” shape, with a large area enclosed by the curve, and the compression power consumption decreased more.

In summary, in water-spray-cooled quasi-isothermal compression, we indirectly controlled the change in the pressure that affected the work of compression by controlling the flow of water-spray. The mass of the water spray was proportional to the heat-transfer area, which directly affected the heat-transfer power. The integral of the flow was the mass, and the integral of the heat-transfer power was the temperature. Therefore, the temperature was obtained by integrating the water-spray flow twice; that is, the change in temperature lagged behind the water spray flow.

Since temperature and pressure were of the same order, the change in pressure (compression work) also lagged behind the water-spray flow. For uniform water spray, the heat-transfer capability of the water spray injected at the later stage of compression was not fully utilized. The improved spray water was injected into the water spray in advance, which increased the heat-transfer time and reduced the compression power consumption.

### 4.2. Impact of Water-Spray Speed

**Figure 11:**
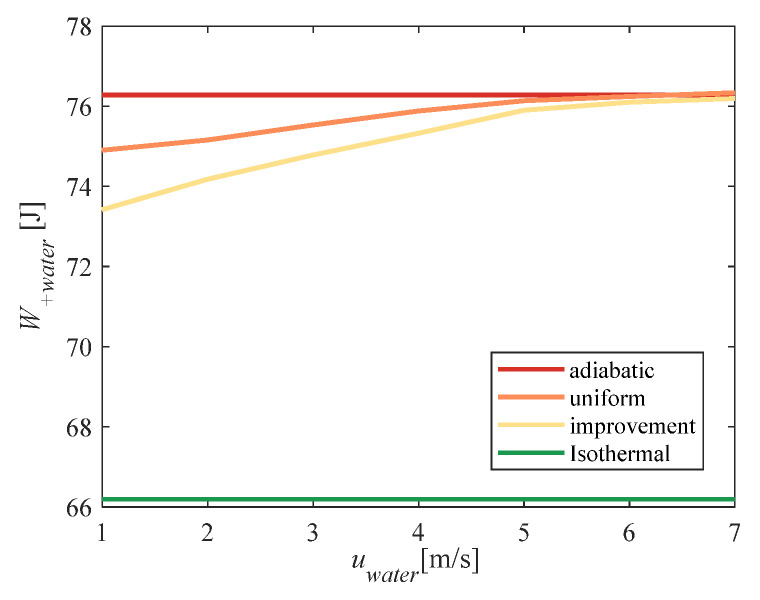
Algorithm verification under different water-spray speeds.

The water-spray speed affected the time at which the water spray moved from the bottom of the compression chamber to the piston. With the decrease in water-spray speed, the heat-transfer time increased so that the heat-transfer capacity of the water spray was more fully utilized during the compression process. Figure 11 shows that the total power consumption decreased with decreasing water spray speed. When the water spray speed was seven times lower than the piston speed, the improvement algorithm could effectively reduce the total power consumption. As the water spray speed decreased, the reduction in total power consumption was more pronounced.

### 4.3. Influence of Rotation Speed

**Figure 12:**
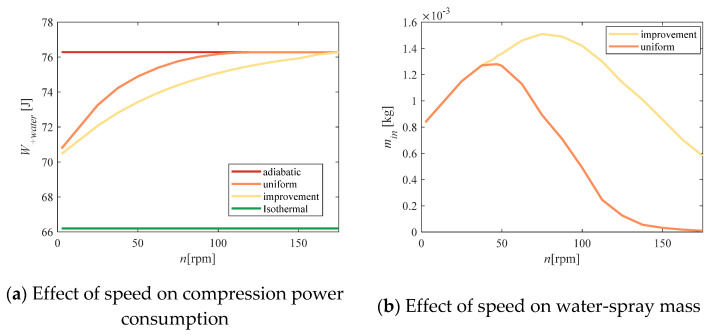
Algorithm verification at different rotation speeds.

The compressor speed affects the compression cycle, as well as the heat-transfer time. Reducing the rotational speed increased the heat-transfer time, which allowed the water-spray to perform more heat exchange. Figure 12 shows that the total power consumption decreased as the speed decreased. When the rotation speed was less than 175 rpm/min, the improvement algorithm could effectively reduce the total power consumption. However, with decreasing rotation speed, the improvement effect became less obvious. When the compression speed was very small, the utilization rate of the water spray increased significantly. At this time, there was almost no difference between the uniform scheme and the improved scheme.

### 4.4. Influence of Water Pressure

**Figure 13:**
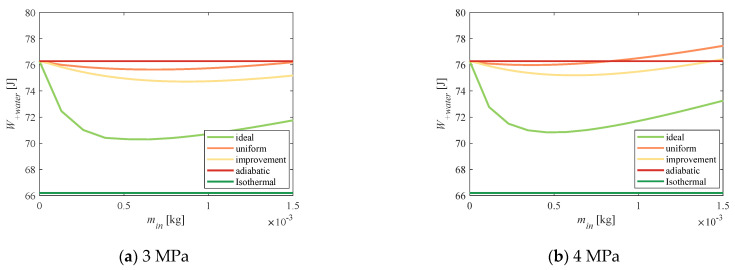

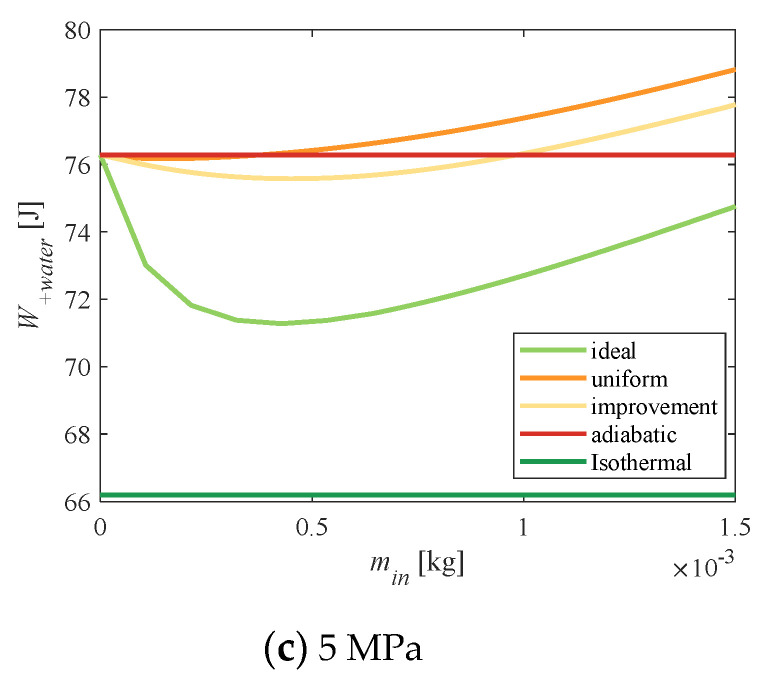
Algorithm verification under different water pressures.

The working pressure of the atomizing nozzle affected both the particle size and the atomized water-spray work. Table 2 lists the measured value of the water-spray particle size in the range of 2~5 MPa. When the water pressure was increased, the particle size of the water spray decreased, and the heat-transfer area increased. Figure 13a–c demonstrate that the above improvement algorithm was still applicable under different water-pressure conditions. As the water pressure rose, the total power consumption increased, and the difference between the improved total power consumption obtained by the improved water-spray scheme and the uniform water-spray scheme was reduced. Moreover, with other conditions determined, when the water-spray pressure reached 5 MPa, the uniform scheme was almost no longer applicable, but the improved power consumption of the improved scheme was still less than the adiabatic power consumption.

## 5. Conclusions

The high proportion of water-atomization energy consumed by the water pump and nozzle system in water-spray-cooled quasi-isothermal compression affected the total power consumption of the compression system. This paper utilized a method to improve water-spray-cooled quasi-isothermal compression by changing the water-spray flow. Based on the compression work and heat-transfer matching, a sequence of water-spray flows (time-varying) was constructed that realized the approximation of the compression process to isothermal compression. According to the minimum total power consumption, the water-spray flow sequence was filtered to obtain improved flow. In comparison with the uniform-flow water spray commonly used in engineering, the following conclusions were obtained:

(1) The water-spray flow of the improved scheme was larger than that of the uniform scheme in the early stage, but decreased and approached 0 in the later stage. The cumulative mass in the compression chamber was the integral of the flow. The cumulative mass of the improved scheme rose fast, and the peak value was high. Later, it decreased and approached 0. However, the uniform scheme still had some remaining mass in the later stage.
(2) Analysis of the dynamics of the compression system revealed that temperature and pressure lagged behind changes in the water-spray flow. Compared with uniform water spray, the effective water-spray quality in the heat-exchange process increased due to the injection of water spray in advance, which could effectively reduce the total power consumption of the compression system.
(3) The water-spray speed and the compressor speed were both related to the heat-transfer time, which affected the performance of the improvement algorithm. When the water-spray speed was lower than the piston speed by seven times, the improvement algorithm could effectively reduce the total power consumption.
(4) The working pressure of the atomizing nozzle was related to the power consumption of the atomization, which affected the effect of the improvement algorithm, and reducing the working pressure made the improvement effect more obvious.

## Figures and Tables

**Table 1:** The ratio of pump energy consumption to compressor energy consumption.

Authors	Jia et al. [16]	Patil et al. [20]
Compression speed (rpm)	60	12
Diameter (μm)	10–100	-
Injection pressure	5 MPa	483 kPa
Flow rate	0.416 g/cycle	1.25 L/min
Generated work/compression work	19.4%	59.51%
Isothermal efficiency	81.7%	95%

**Table 2:** Compressor parameters.

Parameter Name	Symbol	Value
Compression chamber diameter	*D*/m	0.1
Compression chamber length	*L*/m	0.12
Compression chamber volume	*V*_0_/m^3^	9.36 × 10^−4^
Compression ratio	Γ	2~6
Rotating speed	n/rpm	5~90
Water density	*ρ*_water_/kg/m^3^	1000
Initial air temperature	T_0_/K	293
Initial air pressure	*p*_0_/Pa	1 × 10^5^
Heat transfer coefficient	*h*/J/(m^2^·K)	35

**Table 3:** Measurement values of water-spray parameters.

Nozzle Diameter (mm)	Water-Spray Particle Sizes under Different Water Pressures (μm)			
	2 MPa	3 MPa	4 MPa	5 MPa
0.4	28.7	23.3	20.9	19.6
0.6	32.6	28.4	26.2	23.5
0.8	38.5	34.9	31.2	27.3

**Table 4:** Simulation parameters.

Section	Relaxation Factor	Compression Ratio	Water-Spray Speed (m/s)	Water Pressure (MPa)	Rotating Speed (rpm)
3.4	0.0005~1	2	0.4	2	50
3.5, 4.1	0.001	2	0.4	2	50
4.2	0.001	2	0.2~5	2	50
4.3	0.001	2	0.1~0.7	2	25~175
4.4	0.001	2	0.4	2~5	50
